# Polycombing *Drosophila* for Dynamic Developmental Footprints

**DOI:** 10.1371/journal.pbio.0040177

**Published:** 2006-04-20

**Authors:** Emma Hill

In some ways, the developmental passage of a fruit fly from a ball of cells through discrete morphological stages—embryo, pupa, and larva—to a fully formed organism really showcases the potential of biology. Organism development (in this case of a fly) requires a temporally synchronized regulation of a myriad of genes and pathways across the *Drosophila* genome.

No single component of these developmental processes can be considered more important than another—a mistake anywhere can prove fatal. One set of participants in *Drosophila* development are the Polycomb group (PcG) proteins, which bind to regions of chromatin (the genomic DNA twisted around histone proteins that helps structure the chromosomes) and silence the transcription of nearby genes. A handful of PcG response elements (PREs—the regions of the genome where PcGs bind) have been characterized to date, but until now not much was known about their broader distribution across the genome. Nicolas Nègre, Giacomo Cavalli, and colleagues provide just such a map of PcG binding locations across chromosomes X and 2 of *Drosophila melanogaster*. They observe a wide distribution that varies from one developmental stage to the next.

PcG proteins actually form multiprotein complexes, one of which—the PRC1 complex—contains two specific proteins, PC and PH. PC and PH have in the past been seen to co-localize with another protein called the GAGA-factor, or GAF. Nègre and colleagues mapped the binding locations of individual PC, PH, and GAF proteins (separately) to DNA from embryos, pupae, and adult male and female flies. They did this with a technique called “ChIP on chip,” which identifies matches between the region of chromatin where the protein of interest has bound (via chromatin immunoprecipitation, or ChIP) and the corresponding genomic region on a tiled microarray (or chip).

To double-check that their matches were sensible, some known PREs were placed on the microarrays (acting as positive controls), along with some randomly selected DNA fragments unrelated to any known PREs (negative controls). To confirm that the chromosomal locations of the ChIP-on-chip matches corresponded with the correct region of the *Drosophila* genome, the authors also undertook some fluorescence mapping experiments and, reassuringly, observed perfect co-localization of sites mapped by ChIP on chip with sites mapped on the chromosomes by cytology (observing the band on the chromosome that fluoresces).

So how do these proteins distribute over the genome? As expected, PC and PH showed almost identical distribution, which confirms their presence together in the PRC1 complex. GAF binding did not overlap perfectly with the PRC1 proteins and actually bound to more sites in total. GAF also tended to bind to narrower chromatin regions, while PC and PH were more often spread over larger regions. These results indicate that PC and PH binding interactions do not depend on GAF.

Interestingly, the PRC1 proteins frequently bound in clusters, and so it seems that in some genomic regions, more than one PRE must exist in proximate locations. The genes nearest to the PREs, as expected, are most frequently biased toward regulatory and developmental functions. Their distances from the PREs, however, are variable, and it is likely that the PcGs must use a variety of mechanisms to turn off transcription of these nearby genes (perhaps by binding on top of the genes' promoter region, or maybe by linking multiple PREs to interfere with more distant promoters).

How did the binding of these proteins differ across the different developmental time points? While the positions of GAF localization remained quite constant, the PcG protein binding sites did not. Although some PREs were maintained at all times, other PcG protein locations varied considerably, implying that PcGs employ dynamic regulation over time. In fact, adult male flies had the most divergent profile of PREs, suggesting that some male-specific development might be regulated by these PcG proteins.

Nègre, Cavalli, and colleagues show that these PcG proteins do indeed constitute a critical component of *Drosophila* development and are likely to instruct a variety of developmental processes through their regulation of genes across the genome. The next exciting steps in better understanding the molecular construction of a fly will be to extend this analysis to the remaining *Drosophila* chromosomes, across other developmental stages, and in different tissue types. From there, scientists can similarly probe what makes a mammal, and the extent to which these mechanisms are conserved across distant species.

## 

**Figure:**
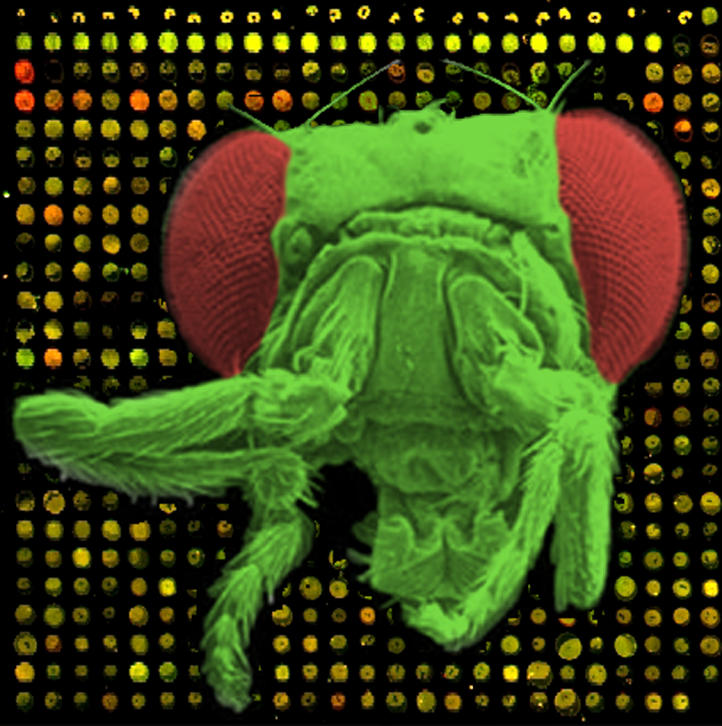
Mapping the chromosomal distribution of Polycomb group proteins in *Drosophila* to study the developmental maintenance of cell identity.

